# Vulvar Metastasis in Renal Cell Carcinoma: A Case Report Highlighting the Aggressive Nature of Clear Cell Renal Cell Carcinoma

**DOI:** 10.3390/curroncol32010004

**Published:** 2024-12-25

**Authors:** Andreea Boiangiu, Ana-Maria Cioca, Gabriel-Petre Gorecki, Romina-Marina Sima, Liana Pleș, Marius-Bogdan Novac, Ionut-Simion Coman, Valentin-Titus Grigorean, Vasile Lungu, Mihai-Teodor Georgescu, George-Alexandru Filipescu

**Affiliations:** 1 Carol Davila University of Medicine and Pharmacy, 020021 Bucharest, Romania; andreea.boiangiu@umfcd.ro (A.B.); romina.sima@umfcd.ro (R.-M.S.); liana.ples@umfcd.ro (L.P.); ionut.coman@umfcd.ro (I.-S.C.); valentin.grigorean@umfcd.ro (V.-T.G.); mihai.georgescu@umfcd.ro (M.-T.G.); alexandru.filipescu@umfcd.ro (G.-A.F.); 2 Department of Obstetrics and Gynecology, Elias University Hospital, 011461 Bucharest, Romania; anamariacioca875@gmail.com; 3 Department of Anesthesia and Intensive Care, Faculty of Medicine, “Titu Maiorescu” University, 031593 Bucharest, Romania; 4 Department of Anesthesia and Intensive Care, CF2 Clinical Hospital, 011464 Bucharest, Romania; 5 Department of Obstetrics and Gynecology, The “Bucur” Maternity, “Saint John” Hospital, 040294 Bucharest, Romania; 6 Department of Anesthesiology and Intensive Care, University of Medicine and Pharmacy, 200349 Craiova, Romania; marius.novac@umfcv.ro; 7 Department of Surgery, Bagdasar-Arseni Clinical Emergency Hospital, 041915 Bucharest, Romania; 8 Department of General Surgery, CF2 Clinical Hospital, 011464 Bucharest, Romania; vasilelungu.md@gmail.com; 9 “Prof. Dr. Al. Trestioreanu” Oncology Discipline, “Carol Davila” University of Medicine and Pharmacy, 020021 Bucharest, Romania

**Keywords:** vulvar cancer, clear cell renal carcinoma, metastasis, vulvar metastasis, kidney tumor

## Abstract

Vulvar cancer is one of the rarest gynecological malignancies. The development of this condition can be associated with either dysplasia linked to human papillomavirus (HPV), primarily affecting younger women, or vulvar dermatoses such as lichen sclerosus, which predominantly affect older women. Over the last decade, the incidence of vulvar cancer has risen by 0.6% annually, while the relative survival rate has declined. Although metastasis to the vulva is uncommon, it can occur, particularly from cancers in nearby organs such as the cervix, bladder, rectum, or anus. More rarely, metastases from breast cancer and renal cell carcinoma have been reported in the vulva. Vaginal metastases from clear cell renal carcinoma are especially rare. In this article, we present the case of a 56-year-old patient diagnosed with clear cell renal carcinoma, who came to our clinic with a lesion on the right labia, which was identified as a metastasis originating from the kidney. Given the rarity of genital metastases in renal cancer, such cases should be examined and discussed to encourage further research and studies.

## 1. Introduction

Metastases to the vulva are rare but can occur, especially from cancers originating in nearby organs such as the cervix, bladder, rectum, or anus [1,2]. The most common primary cancers that metastasize to the vulva include those from the lower genital tract (like cervical cancer) and gastrointestinal cancers [3]. Less frequently, breast cancer and renal cell carcinoma have also been reported to metastasize to the vulva [4].

Clear cell renal carcinoma (RCC) constitutes 70% of all epithelial renal tumors, and its incidence is on the rise [5]. The majority of cases are diagnosed in adults over the age of 40 [6]. The incidence is higher among the black population compared to white people in North America (Chow [7,8]). Apart from major hereditary conditions (such as Von Hippel–Lindau disease and tuberous sclerosis), smoking is considered the primary risk factor for the development of RCC [9]. Other factors associated with RCC include obesity, particularly in women, and various chemical agents [6,9]. Although the long-term use of phenacetin and acetaminophen has been linked to the occurrence of renal carcinoma, the former is much more strongly associated with transitional cell carcinoma of the renal pelvis. RCC has also been reported in association with AIDS [6].

RCC commonly metastasizes to the lungs and bones, but vaginal metastases have also been described [10]. A previous review of 85 cases of RCC with vaginal metastases reported a median overall survival of 19 months for patients with synchronous or metachronous metastases [11].

Despite the rarity of vulvar metastases from renal cell carcinoma, these cases highlight the aggressive nature of the disease and the challenges in its management. Furthermore, controversial hypotheses regarding the routes of spread (e.g., retrograde venous versus lymphatic pathways) continue to be debated in the literature. This case emphasizes the need for heightened clinical awareness and the detailed evaluation of atypical metastatic sites. Our study aims to examine a rare case of vulvar metastasis from clear cell renal carcinoma, providing insights into the diagnostic process, treatment approach, and clinical outcomes.

## 2. Case Description

The patient, a 56-year-old woman with a complex personal medical history, including one childbirth and two abortions, had been in menopause since the age of 49; additionally, the patient was known to have multiple cardiovascular pathologies, which could have potentially complicated her clinical picture. These included arterial hypertension, chronic venous insufficiency, and mitral insufficiency. These comorbidities necessitated careful consideration during both the diagnostic process and treatment planning.

She presented to the hospital with complaints of pain localized in the upper abdominal region. Based on her symptoms, an abdominal ultrasound was performed, which revealed a relatively well-defined, round-to-oval mass measuring approximately 69 by 61 mm, located in the left flank.

**Figure 1:**
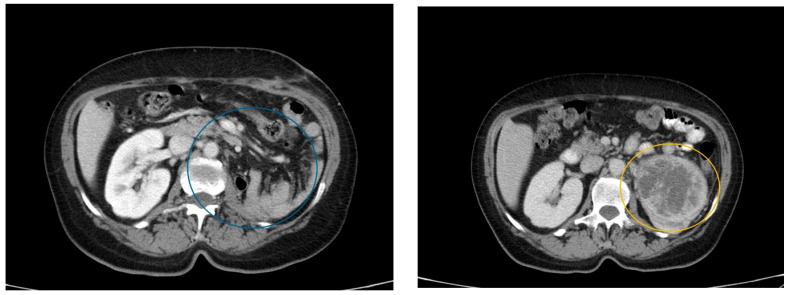
CT scan revealing the tumor formation in the left kidney (marked in blue and yellow circles).

Following recommendations, one month later, she underwent a CT scan to complete diagnostic imaging (Figure 1). The scan showed a macronodular tumor in the left kidney, with dimensions of approximately 70/81/86 mm. The tumor appeared heterogeneous, with a predominantly peripheral, solid, and iodophilic component and a central, non-homogeneous, hypodense, and semi-solid component, located in the lower half of the anterior valve. The lesion exerted compressive effects on the pyelocaliceal system and renal pedicle, resulting in Grade II ureterohydronephrosis and the dilation of the ipsilateral renal vein, with extracapsular extension beyond the anterior perirenal fascia. Regional lumbar aortic lymph nodes with diameters up to 7/12 mm were also noted.

Based on these imaging findings, a suspicion of left renal neoplasm was raised, and two weeks later, a laparoscopic left nephrectomy was performed. The histopathological examination of the resected specimen confirmed the diagnosis of clear cell renal carcinoma, Fuhrman grade G3, with focal infiltration of perirenal adipose tissue and tumor necrosis present (60% of the tumor mass). The tumor was staged at pT3aN0.

**Figure 2:**
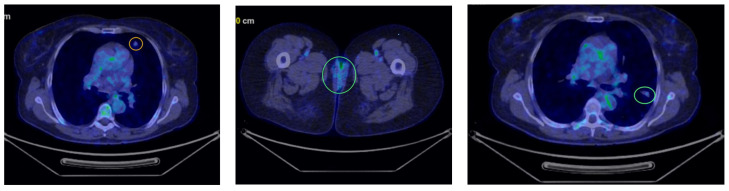
First PET-CT revealing multiple bilateral non-calcified pulmonary nodules (marked in yellow and green circles).

**Figure 3:**
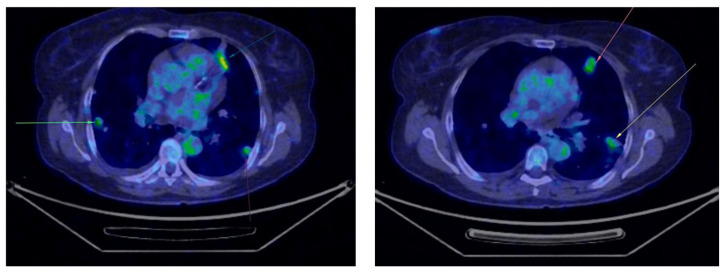
Second PET-CT scan showing the progression of the lung lesions (marked with arrows).

Five month later, the patient underwent her first follow-up PET-CT, which revealed multiple bilateral non-calcified pulmonary nodules, most of which showed minimal uptake, raising suspicion of secondary metastases (Figure 2). In the follow-up PET-CT performed six months later, multiple non-calcified nodules were observed bilaterally in the pulmonary parenchyma, with progressive dimensional and metabolic increases, the largest measuring 28/20 mm. These nodules were biopsied. The preliminary histopathological examination suggested carcinomatous infiltration, which was confirmed by the final histopathological report as clear cell carcinoma with pulmonary localization, indicative of secondary metastasis (Figure 3).

The lung specimen sent for pathological examination underwent immunohistochemical analysis. The tumor cells were positive for PAX8 and CD10, negative for CK7 and S100, with a Ki67 expression of 15–20%.

The patient presented to our clinic one year later with a tumor on the right labium, initially resembling a lipoma, measuring approximately 3/2 cm. The tumor was resected, and the histopathological report indicated a secondary determination of clear cell renal carcinoma (an area with abundant hemorrhagic infiltration and tumor proliferation composed of large/medium-sized cells with abundant, clear cytoplasm, round–oval nuclei arranged in groups and acinar structures, with sclerohyaline stroma).

The patient began oncological treatment consisting of five sessions of immunotherapy with Axitinib and Bavencio, along with fifteen sessions of external irradiation using the IMRT-VMAT technique, targeting the right labial tumor.

**Figure 4:**
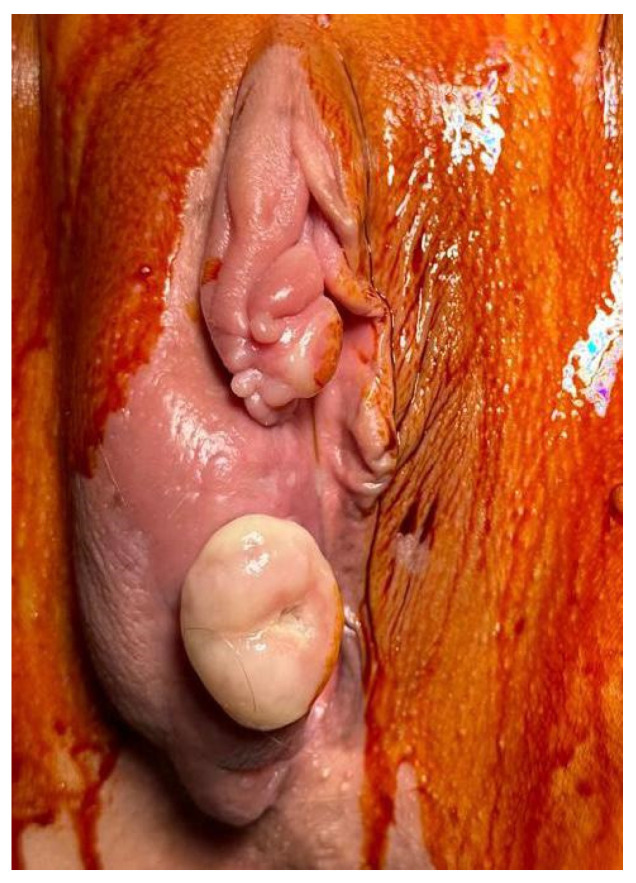
Vulvar mass.

**Figure 5:**
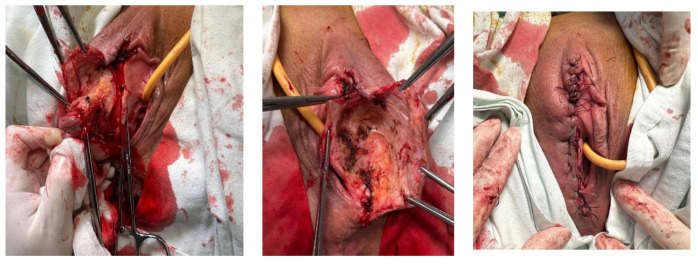
Intraoperative images from the excision of the vulvar mass.

Two months later, the patient returned to our clinic for the progression of the vulvar tumor (Figure 4). Excision of the mass was performed (Figure 5), along with the dissection of the right inguinal lymph nodes, and the samples were sent for histopathological examination. The histopathological report confirmed a secondary determination of clear cell renal carcinoma, with no neoplastic infiltrates found in the examined lymph node sections.

**Figure 6:**
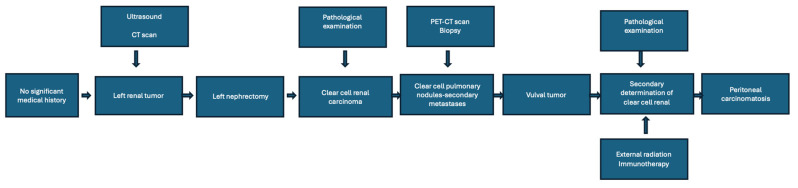
Flow diagram of the patient medical history.

After three months, the patient’s general condition deteriorated, and, at the time of examination, there was suspicion of intestinal obstruction. At this stage, the patient had secondary metastases in the brain, liver, bones, and vulva. Surgery was considered for the suspected intestinal obstruction, and, intraoperatively, a tumor block with peritoneal carcinomatosis was discovered, severely limiting surgical options. The patient’s condition rapidly deteriorated, and she passed away within two weeks, two years from her first admission to our department for the renal tumor. For a more comprehensive view of the medical history, we created a flow diagram (Figure 6).

## 3. Discussion

Worldwide, renal cancer is the seventh most common cancer in men and the tenth in women [12,13]. Clear cell renal carcinoma (RCC) represents 3% of all adult cancers and accounts for 85% of primary kidney tumors [14,15]. It is the most prevalent type of renal carcinoma and the second most frequent urological cancer. About 30% of RCC cases develop metastases, which can spread through the lymphatic system, the bloodstream, and direct invasion or transcoelomically [12,13,16]. These metastases most commonly affect the lungs, bones, adrenal glands, liver, lymph nodes, and brain, although they can also, though much less frequently, spread to the thyroid, orbit, nasal structures, vagina, gallbladder, pancreas, sublingual tissues, and soft tissues of the extremities [17,18]. Metastases can be synchronous (18%) or metachronous (50%) [19]. Vaginal metastases from RCC are exceptionally rare, and it is even less common for RCC to initially present as a vaginal tumor. When RCC metastasizes to the vagina, the lesion is usually solitary and located in the lower third of the vaginal wall [20,21]. Tumors that metastasize to the vagina most often originate from the left kidney, with tumor emboli traveling through the left renal vein into the ovarian vein and uterovaginal plexus [22].

The first case of RCC-related vaginal metastasis was reported by Penham in 1906 [23]. Since then, fewer than 100 such cases have been recorded, with RCC initially presenting as vaginal metastasis in only 3 of these cases [13].

Vaginal metastases originating from clear cell renal carcinoma (RCC) are uncommon. A review of 85 cases found a median age at diagnosis of 57 years (ranging from 14 to 88 years), with 65% of patients presenting with symptoms such as vaginal discharge, bleeding, or a mass effect. Vaginal lesions varied in size from 0.5 to 8 cm, and these metastases typically occurred before the diagnosis of RCC, with only a few cases appearing afterward. In 63% of instances, the primary tumor was located in the left kidney, and the vaginal metastases were usually solitary, located on the same side as the primary tumor, often in the lower third of the anterior vaginal wall [10,11,20]. The presence or absence of other secondary tumors is the most critical prognostic factor for patients with vaginal metastases. Metachronous metastases tend to be associated with a longer survival than synchronous ones, with an overall median survival of 19 months (ranging from 1 to 96 months) [10,24,25]. Although vaginal metastases from RCC can spread through urinary, lymphatic, or systemic pathways, the only confirmed route is retrograde venous spread [26,27].

Immunotherapy represents a major advancement in the treatment of clear cell renal carcinoma by harnessing the immune system to combat tumor cells. Modern approaches focus on immune checkpoint inhibitors, cytokine therapies, and targeted combination treatments [28]. Checkpoint inhibitors, such as antibodies targeting PD-1/PD-L1 and CTLA-4, block immune-suppressive pathways, reactivating T-cells and restoring antitumor immunity [28,29,30].

While older cytokine therapies like interleukin-2 and interferon-α had limited success due to significant toxicity, their use has declined in favor of more effective treatments. A notable progress is the combination of Axitinib (a VEGFR inhibitor) and Avelumab (a PD-L1 inhibitor), which simultaneously inhibits tumor angiogenesis and enhances the immune response. This dual mechanism has demonstrated improved clinical outcomes, including prolonged progression-free survival, positioning it as a valuable strategy in the management of clear cell renal carcinoma [28].

## 4. Conclusions

Vulvar metastases from clear cell renal carcinoma are exceedingly rare and pose significant diagnostic and therapeutic challenges. This case underlines the aggressive behavior of clear cell renal carcinoma and the importance of early detection and multidisciplinary management. Further studies are needed to optimize therapeutic strategies for patients with atypical metastatic presentations.

## Data Availability

Data availability by contacting the corresponding author.

## References

[1] Weinberg D., Gomez-Martinez R.A. (2019). Vulvar Cancer. Obstet. Gynecol. Clin. N. Am..

[2] Dellinger T.H., Hakim A.A., Lee S.J., Wakabayashi M.T., Morgan R.J., Han E.S. (2016). Surgical Management of Vulvar Cancer. J. Natl. Compr. Cancer Netw..

[3] Pliszka A., Rajda S., Wawrzyniak A., Walocha J., Polguj M., Wysiadecki G., Clarke E., Golberg M., Zarzecki M., Balawender K. (2023). Testicular Metastasis from Renal Cell Carcinoma: A Systematic Review. J. Clin. Med..

[4] Giannini A., D’Oria O., Chiofalo B., Bruno V., Baiocco E., Mancini E., Mancari R., Vincenzoni C., Cutillo G., Vizza E. (2021). The giant steps in surgical downsizing toward a personalized treatment of vulvar cancer. J. Obstet. Gynaecol. Res..

[5] Siegel R.L., Miller K.D., Jemal A. (2016). Cancer statistics, 2016. CA Cancer J. Clin..

[6] Grignon D.J., Che M. (2005). Clear cell renal cell carcinoma. Clin. Lab. Med..

[7] How J.A., Jazaeri A.A., Soliman P.T., Fleming N.D., Gong J., Piha-Paul S.A., Janku F., Stephen B., Naing A. (2021). Pembrolizumab in vaginal and vulvar squamous cell carcinoma: A case series from a phase II basket trial. Sci. Rep..

[8] Mousavi S.E., Najafi M., Aslani A., Fazlollahi A., Yekta Z., Sadri M., Nejadghaderi S.A. (2024). A population-based study on incidence trends of kidney and renal pelvis cancers in the United States over 2000–2020. Sci. Rep..

[9] Moyad M.A. (2001). Review of potential risk factors for kidney (renal cell) cancer. Semin. Urol. Oncol..

[10] Hisano M., Kato R., Itamochi H., Matsuura T., Maekawa S., Kato Y., Kanehira M., Takata R., Baba T., Obara W. (2020). Rapid progression of recurrent disease in a patient with renal cell carcinoma with vaginal metastasis. IJU Case Rep..

[11] Mendese G.W., Ayvazian P.J., Li C. (2006). Renal cell carcinoma presenting as a perineal mass: Case report and review of the liter-ature. Urology.

[12] Escudier B., Porta C., Schmidinger M., Rioux-Leclercq N., Bex A., Khoo V., Grünwald V., Gillessen S., Horwich A., ESMO Guidelines Committee (2019). Renal cell carcinoma: ESMO Clinical Practice Guidelines for diagnosis, treatment and follow-up. Ann. Oncol..

[13] Yordanov A., Kostov S., Kornovski Y., Ivanova Y., Slavchev S., Kostov G., Strashilov S. (2022). Initial presentation of renal cell carcinoma as a vaginal mass with excessive bleeding. Menopausal Rev..

[14] Ouellet S., Binette A., Nguyen A., Garde-Granger P., Sabbagh R. (2018). Metastatic renal cell carcinoma initially presenting with hematochezia and subse-quently with vaginal bleeding: A case report. BMC Urol..

[15] Geng Z., Zhang Q., Jia P., Miao J., Lin Q. (2022). Severe vaginal bleeding due to vaginal metastasis from renal cell carcinoma with inferior vena cava tumor thrombus: A case report. Medicine.

[16] Pernicone E., Fabrega-Foster K. (2023). Clinically Silent, Metastatic Renal Cell Carcinoma Detected on Routine Screening Mammogram: A Report of a Rare Case and Review of Literature. Cureus.

[17] Jimenez A.R., Rolon M.d.M.R., Eyzaguirre E., Clement C. (2018). Vaginal bleeding as initial presentation of an aggressive renal cell carcinoma: A case report and review of the literature. Case Rep. Pathol..

[18] Sadler G.J., Anderson M.R., Moss M.S., Wilson P.G. (2007). Metastases from renal cell carcinoma presenting as gastrointestinal bleeding: Two case reports and a review of the literature. BMC Gastroenterol..

[19] Brufau B.P., Cerqueda C.S., Villalba L.B., Izquierdo R.S., González B.M., Molina C.N. (2013). Metastatic renal cell carcinoma: Radiologic findings and assessment of response to targeted antiangiogenic therapy by using multidetector CT. RadioGraphics.

[20] Benbrahim Z., Chouaib A., Mazeron R., Leger-Ravet M.B., Lefort C., Lhommé C., El Mesbahi O., Escudier B. (2013). Gynecologic bleeding revealing vaginal metastasis of renal cell carcinoma. Pan Afr. Med. J..

[21] Rehailia-Blanchard A., Morel A., Rancoul C., He M.Y., Magné N., Falkowski S. (2018). Vaginal metastasis of renal clear-cell cancel. Gulf. J. Oncol..

[22] Allard J.E., McBroom J.W., Zahn C.M., McLeod D., Maxwell G.L. (2004). Vaginal metastasis and thrombocytopenia from renal cell carcinoma. Gynecol. Oncol..

[23] Peham I. (1906). Hypernephrom der linken Niere mitein er Metastase in der Vagina. Zentralbl. Gynak. Ol..

[24] Xu Y., Hou R., Lu Q., Deng Y., Hu B. (2017). Renal clear cell carcinoma metastasis to the breast ten years after nephrectomy: A case report and literature review. Diagn. Pathol..

[25] Abu-Rustum N.R., Yashar C.M., Arend R., Barber E., Bradley K., Brooks R., Campos S.M., Chino J., Chon H.S., Crispens M.A. (2024). Vulvar Cancer, Version 3.2024, NCCN Clinical Practice Guidelines in Oncology. J. Natl. Compr. Cancer Netw..

[26] Ruiz J.M., Mondéjar R.R., López P.C., Sanchiz C.M., Moreno M.J.D., Navarro H.P., Teruel M.P., Rodríguez J.A.V. (2011). Vaginal metastasis of a clear renal cell carcinoma. Arch. Esp. Urol..

[27] Ladjevic I.L., Stefanovic A., Kadija S., Terzic M., Jeremic K., Janjic T. (2016). Vagina as a rare location of renal cell carcinoma metastasis. Eur. J. Gynaecol. Oncol..

[28] Peinemann F., Unverzagt S., Hadjinicolaou A.V., Moldenhauer I. (2019). Immunotherapy for metastatic renal cell carcinoma: A systematic review. J. Evid.-Based Med..

[29] Cho Y.H., Kim M.S., Chung H.S., Hwang E.C. (2017). Novel immunotherapy in metastatic renal cell carcinoma. Investig. Clin. Urol..

[30] Atkins M.B., Clark J.I., Quinn D.I. (2017). Immune checkpoint inhibitors in advanced renal cell carcinoma: Experience to date and future directions. Ann. Oncol..

